# 4MRNA: a new approach for nucleic acid molecular replacement using models with diverse parameter patterns

**DOI:** 10.1261/rna.081112.126

**Published:** 2026-10

**Authors:** Shin Ando, Jiro Kondo

**Affiliations:** 1 Graduate School of Science and Technology, Sophia University, Tokyo 102-8554, Japan; 2 Department of Materials and Life Sciences, Faculty of Science and Technology, Sophia University, Tokyo 102-8554, Japan

**Keywords:** three-dimensional structure, X-ray crystallography, molecular replacement, nucleic acid

## Abstract

Structural analysis of nucleic acids lags behind that of proteins, partly because most fundamental structural analysis techniques have been primarily developed for proteins. The molecular replacement (MR) method, commonly used for phase determination in protein crystallography, encounters unique challenges when applied to nucleic acids. Nucleic acids can have different three-dimensional structures even with the same sequence, which often render database entries or predicted models unsuitable as search models for MR. To overcome the limitation, we developed a novel strategy termed 4MRNA, which stands for Massive Multi-type Model Molecular Replacement for Nucleic Acids. This method introduces a new principle for MR, which is the systematic creation of diverse search models through parameter adjustment. By identifying the parameters that critically influence MR and generating models based on their statistical analysis, 4MRNA can provide search models that closely approximate target structures and thereby improve the success rate of MR. Its effectiveness was validated across comprehensive test cases including canonical duplexes, duplexes with bulges and internal loops, the more complex structure of transfer RNA, and a previously unreported DNA structure. 4MRNA is anticipated to become an indispensable tool for nucleic acid structure determination, profoundly advancing fundamental research and extending its impact to wide-ranging applications including structure-based drug design and nucleic acid nanotechnology.

## INTRODUCTION

The detailed observation of molecular three-dimensional structures facilitates the rational design of pharmaceuticals. This approach, called structure-based drug design (SBDD), has already been successfully applied to the development of small-molecule drugs (Kondo 2012; Hardianto et al. 2019; Bajad et al. 2021). Moreover, SBDD is expected to play a crucial role in the development of nucleic acid therapeutics as a new modality, including antisense oligonucleotides, small interfering RNAs, and aptamers (Egli and Manoharan 2023). Additionally, studying nucleic acid structures has applications in DNA and RNA nanotechnology, such as the design of self-assembling nanostructures (Zheng et al. 2009), functional nucleic acid materials (Kondo et al. 2017; Ono et al. 2019), and fluorescence-based imaging systems (Trachman et al. 2017; Cerretani et al. 2019; Miyauchi et al. 2025).

Given these backgrounds, fundamental research on the structural analysis of nucleic acids is becoming increasingly critical. However, this field has lagged behind protein structure research. This gap is partly because many fundamental structural analysis techniques were originally developed for proteins. Thus, it is necessary to advance these basic techniques specifically tailored for nucleic acids. In this study, we focused on advancing the phase determination process within X-ray crystallography.

In X-ray crystallography, diffraction data are obtained by exposing biomolecular crystals to X-rays. However, the phase information required to construct an electron density map is not directly accessible from the diffraction data. This is a well-known challenge referred to as the phase problem. Molecular replacement (MR) is a widely used method to overcome this issue (Rossmann 1990). It utilizes a search model, typically a homologous structure, which is systematically rotated and translated within the unit cell to find the orientation and position that best match the observed diffraction patterns. Once the optimal placement is identified, the search model provides an initial approximation of the target structure, enabling the estimation of the missing phase information. This initial phase estimate allows for the computation of an electron density map, which serves as the basis for model building and refinement. Through iterative cycles of refinement and model adjustment, the structural model is progressively improved, ultimately leading to an accurate atomic-resolution structure. MR has become an indispensable technique in macromolecular crystallography, particularly when experimental phasing methods such as multiwavelength anomalous diffraction (MAD) or single-wavelength anomalous diffraction (SAD) are not feasible.

While structure prediction tools like AlphaFold (Jumper et al. 2021; Abramson et al. 2024) now generate models with sufficient accuracy for MR in the case of proteins, it is indicated that nucleic acid structure prediction has yet to reach a comparable level of reliability (Bernard et al. 2025). This is because nucleic acids can adopt different three-dimensional structures even with the same sequence (Kondo et al. 2007; Kondo and Westhof 2008; Hanessian et al. 2014), posing a fundamental difficulty for sequence-based prediction algorithms (Kwon 2025).

Additionally, many nucleic acid molecules possess helical structures that extend primarily along a single axis. Therefore, while determining the direction of the helical axis is often straightforward, identifying the correct rotation around this axis can be challenging, even when the search model and target structure differ only slightly (Kondo et al. 2008). Furthermore, even minor variations in helical parameters can lead to significant discrepancies in the overall molecular structure, making MR particularly difficult for nucleic acids. Consequently, finding a highly accurate search model in the Protein Data Bank (PDB) (Berman et al. 2000) is essential. However, this is often difficult because the number of nucleic acid structures registered in the PDB is significantly smaller than that of proteins. Even when an appropriate model is available, traditional MR approaches may still fail due to the intrinsic structural flexibility of nucleic acids. Unlike proteins, where minor conformational differences can often be tolerated, small deviations in helical geometry can disrupt the entire molecular alignment, preventing successful phase determination.

To address these challenges, in this study we propose a novel MR strategy specifically tailored for nucleic acids. Our approach involves creating diverse structural models, extending beyond the repertoire of known structures, by modulating parameters that govern nucleic acid three-dimensional structure. Such diversity increases the probability of obtaining models that approximate the correct structure, thereby improving the success rate of MR and opening the way to solve nucleic acid structures that have remained inaccessible by conventional approaches.

## RESULTS

### Statistics of parameters controlling the three-dimensional structure of nucleic acids

Considering that even slight differences in the three-dimensional structures of nucleic acids can hinder structure determination by MR, it was necessary to identify the factors responsible for such structural diversity. To this end, 12 parameters that control the three-dimensional structures of nucleic acids (Lu and Olson 2003) (referred to as helical parameters in this paper) were analyzed. These parameters include 6 bp parameters (Shear, Stretch, Stagger, Buckle, Propeller, and Opening; referred to as intra-base-pair parameters in this paper) and 6 bp step parameters (Shift, Slide, Rise, Tilt, Roll, and Twist; referred to as inter-base-pair parameters in this paper) (Fig. 1).

**FIGURE 1.**
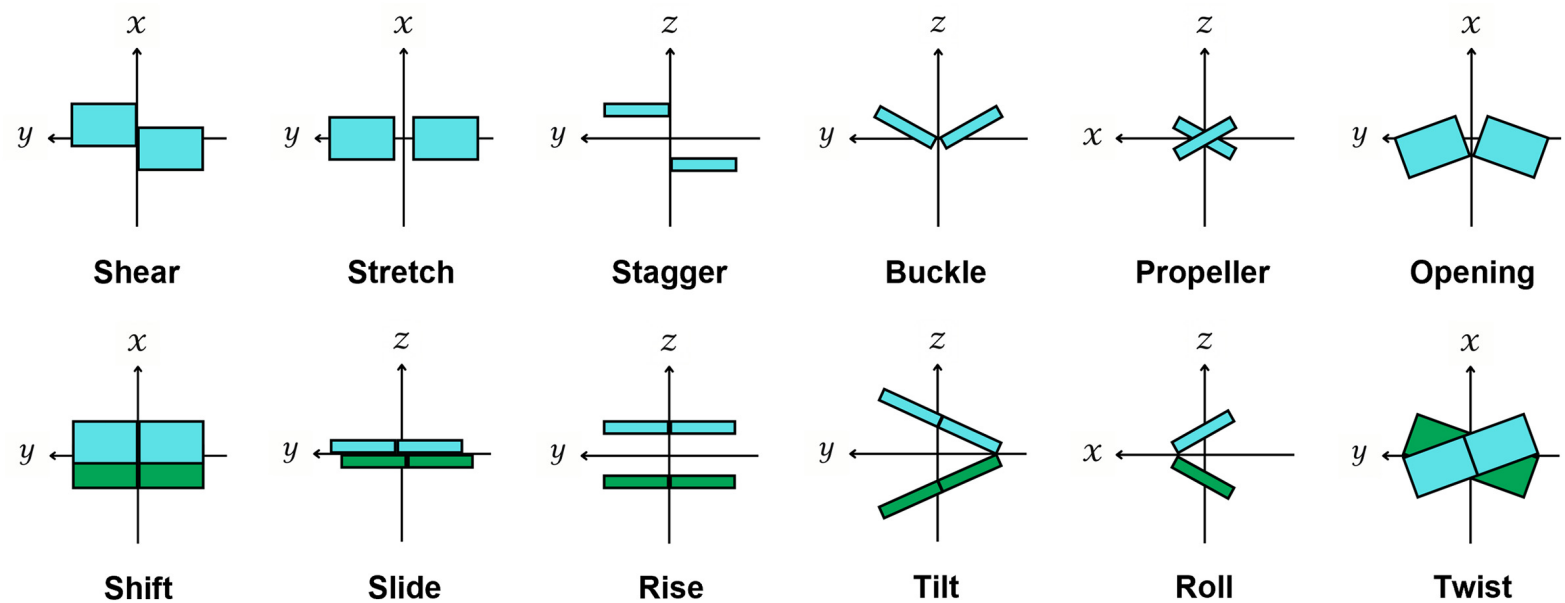
Twelve helical parameters. The upper six are intra-base-pair parameters, which control the length and angle within a base pair. The lower six are inter-base-pair parameters, which control the length and angle between base pairs. The figure was prepared with reference to Lu and Olson (2003).

The analysis targeted all crystal structures of A-form and B-form duplexes deposited in the PDB. For all base pairs within these nucleic acids, the mean, variance, and standard deviation (σ) of the 12 helical parameters were calculated. These statistical values are summarized in Table 1. A larger data set than those used in previous studies (Olson et al. 2001) was analyzed, but the values obtained were found to be comparable to those previously reported. Among the 12 parameters, Buckle, Propeller, Opening, Tilt, Roll, and Twist exhibited greater variance and standard deviation than the others. This finding indicates that these six parameters contribute more substantially to structural diversification and should therefore be the focus of further analysis.

**TABLE 1.** Statistics of helical parameters

Parameter	A-form duplex				B-form duplex			
	Sample size	Mean	Variance	Standard deviation	Sample size	Mean	Variance	Standard deviation
Intra-base-pair								
Shear (Å)	3551	−0.01	0.09	0.29	4575	0.00	0.13	0.36
Stretch (Å)	3657	−0.17	0.02	0.13	4765	−0.16	0.03	0.16
Stagger (Å)	3772	0.02	0.04	0.21	4623	0.12	0.08	0.28
Buckle (°)	3791	−0.08	40.72	6.38	4635	0.89	55.08	7.42
Propeller (°)	3757	−10.73	34.19	5.85	4626	−10.65	59.46	7.71
Opening (°)	3698	0.41	10.61	3.26	4590	−0.36	19.72	4.44
Inter-base-pair								
Shift (Å)	3405	0.01	0.33	0.58	4168	−0.01	0.35	0.59
Slide (Å)	3372	−1.63	0.16	0.40	4150	0.08	0.45	0.67
Rise (Å)	3360	3.24	0.03	0.18	4100	3.31	0.05	0.22
Tilt (°)	3384	0.10	6.85	2.62	4144	−0.81	11.10	3.33
Roll (°)	3393	7.67	26.26	5.12	4159	0.74	29.28	5.41
Twist (°)	3291	31.26	23.84	4.88	4103	35.21	32.76	5.72

### Effect of parameter changes on molecular replacement

The impact of the six parameters (Buckle, Propeller, Opening, Tilt, Roll, and Twist) on MR was evaluated. Structural models were systematically created by adjusting each base pair to one of three levels (−3σ, ±0, +3σ), and MR was performed using these models. As shown in Figure 2, the distributions of LLG and TFZ were broader when Tilt, Roll, and Twist were changed than when Buckle, Propeller, and Opening were modified. The ideal model, in which all parameters were set to their mean values, yielded LLG = 249 and TFZ = 13.3. These values could not be exceeded by alterations in Buckle, Propeller, and Opening, whereas higher values were achieved through adjustments in Tilt, Roll, and Twist. These results indicate that Tilt, Roll, and Twist have a more critical impact on MR performance. Consequently, the analysis could be narrowed from six to three parameters, which became the primary focus of the new strategy.

**FIGURE 2.**
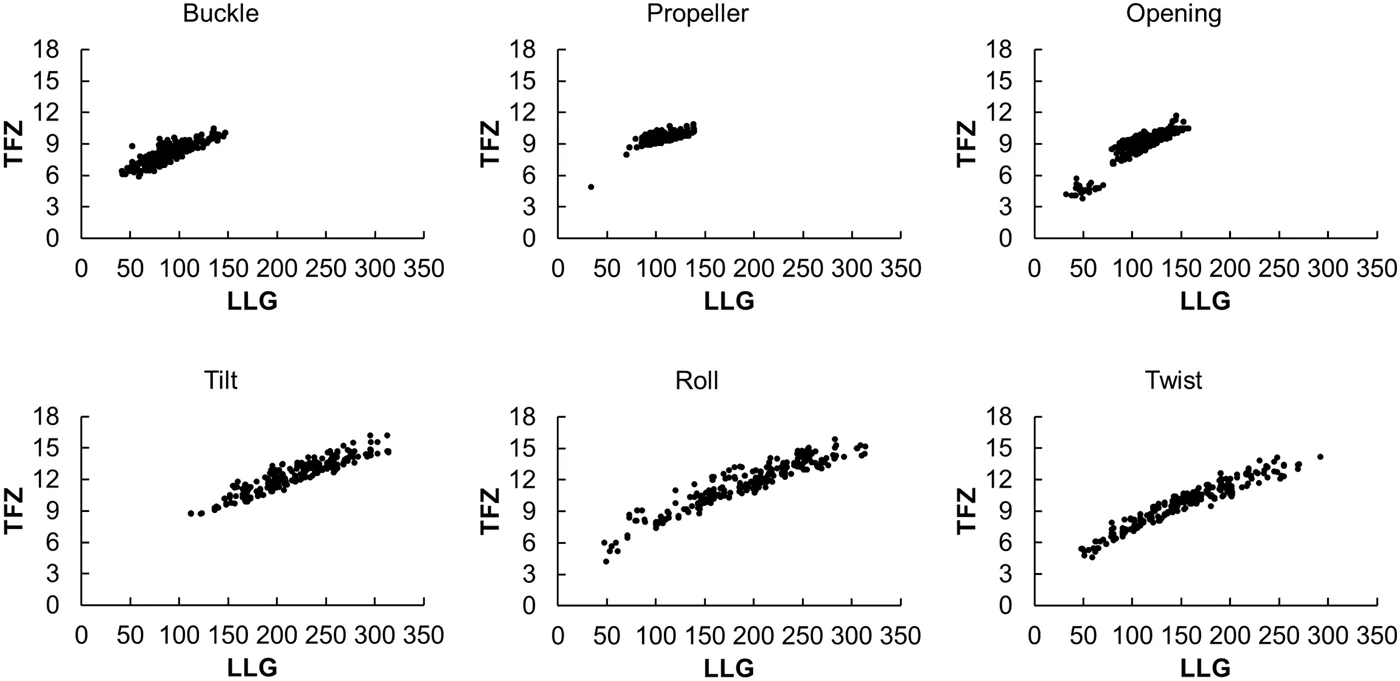
Statistics of MR when six parameters are changed individually. The range of statistical values was broader when adjusting Tilt, Roll, and Twist compared to when adjusting Buckle, Propeller, and Opening.

### Regularity of key helical parameters

Since Tilt, Roll, and Twist were identified as the most influential parameters for MR, the next step was to determine how these parameters should be varied when creating search models under the new strategy. To this end, the regularity of these three parameters was analyzed across all nucleic acid duplex crystal structures. For each duplex, the parameter values were plotted and visualized as graphs (Supplemental Figs. S1–S6).

As representative examples from these data, three nucleic acids with nearly identical sequences but distinct three-dimensional structures are shown in Figure 3. The black and red duplexes adopt similar structures, whereas the blue duplex displays a markedly different structure (Fig. 3A). Their corresponding parameter patterns are shown in Figure 3B. The duplexes with similar structures exhibit closely matching parameter values, while those with different structures show pronounced differences in their parameter values. A common feature across these cases, however, was the presence of mirror-like symmetry in the trajectories of increasing and decreasing values.

**FIGURE 3.**
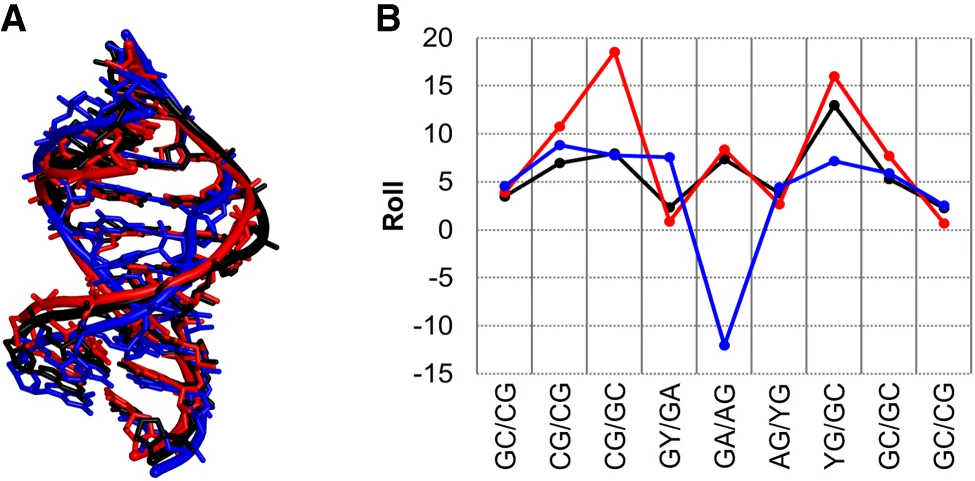
An example of parameter patterns. (*A*) Three structures with almost identical sequences. (Only a single base differs, but the distinction between purine and pyrimidine bases remains consistent.) Black represents PDB ID 5LQO, red represents 5LQT, and blue represents 5LR3 (Huang et al. 2017). The root-mean-square deviation (RMSD) between black and red was 0.761 Å, indicating that they have similar structures. In contrast, the RMSD between black and blue was 2.073 Å, and between red and blue was 2.685 Å, suggesting significant structural differences. (*B*) A graph visualizing the patterns of Roll. The structurally similar black and red exhibit similar patterns, whereas the structurally distinct black/red and blue show markedly different patterns. However, all of their parameter patterns have line symmetry.

Similar symmetric patterns were also observed in many other nucleic acids beyond the examples shown in Figure 3, as summarized in Supplemental Table S1. Although the origin of this symmetry cannot be determined definitively from the present analysis, it may be partly explained by the combined effects of sequence and crystal packing. Self-complementary sequences inherently introduce symmetry at the sequence level. In addition, since most nucleic acid duplexes adopt either terminal-to-terminal packing, in which adjacent duplexes stack along the helical axis, or mid-region-to-terminal packing, in which adjacent duplexes interact with their helical axes arranged nearly perpendicular to each other (Shamim et al. 2020), these packing modes may impose similar structural constraints in both directions from the duplex center. Indeed, the examples shown in Figure 3 exhibit both self-complementary sequences and terminal-to-terminal packing.

These results suggest that even when line symmetry is imposed as a constraint in the new strategy, differences in the overall pattern shape can still allow for reliable discrimination among distinct structures.

### Overview of a new method

In our new strategy, the three helical parameters identified as most influential for MR, Tilt, Roll, and Twist, were systematically varied. After adjusting the parameter values, structural models corresponding to each parameter pattern were constructed and subsequently employed in molecular replacement (Fig. 4A). Each parameter was adjusted using three increments or decrements (–2σ, ±0, +2σ), a choice motivated by the statistical principle that 95.45% of the data falls within ±2σ of the mean under a normal distribution. In other words, most structural diversity can be captured within this range, even in cases of significant structural distortions.

**FIGURE 4.**
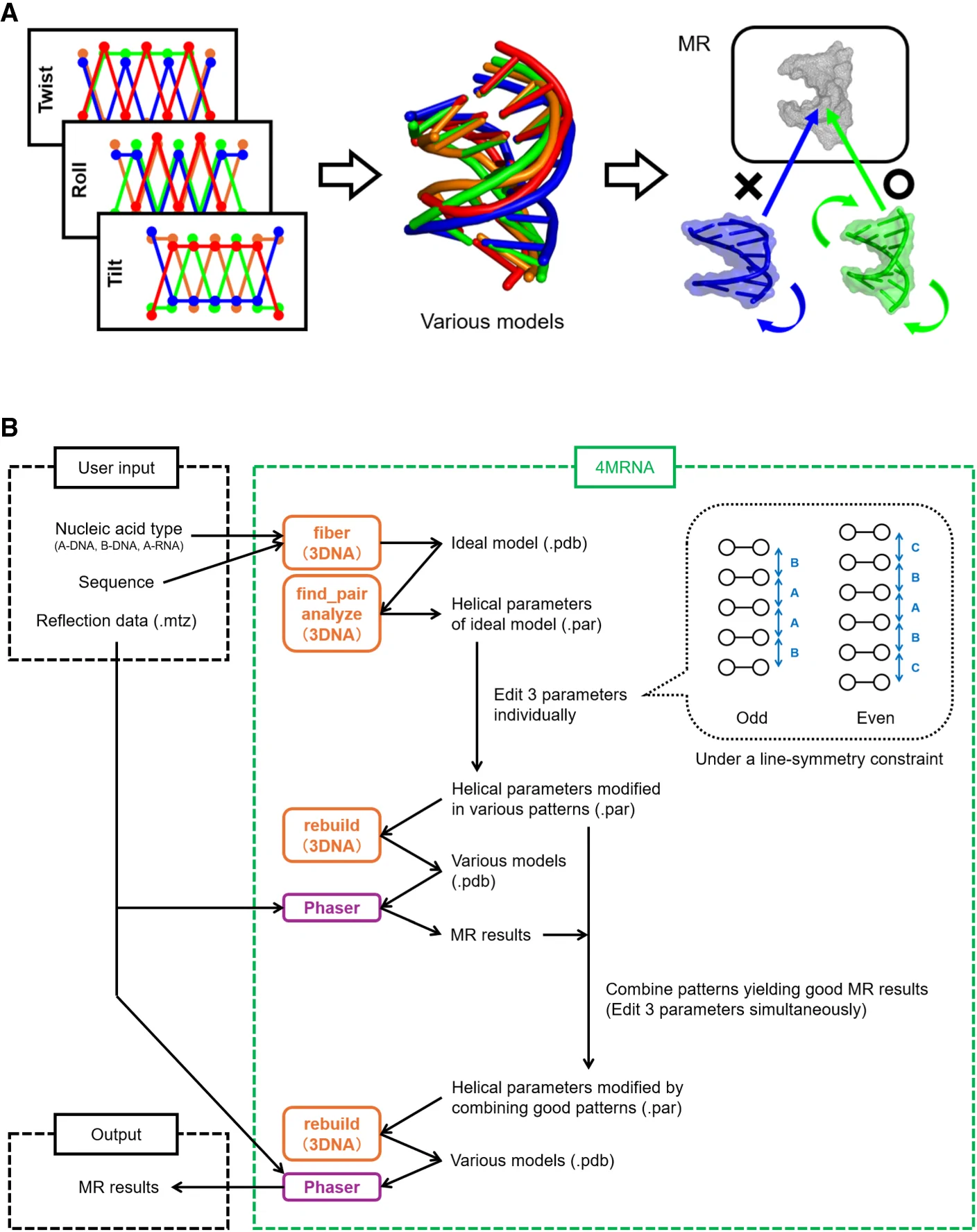
Schematic overview of the new method 4MRNA. (*A*) Conceptual strategy. (*B*) Detailed workflow. In the diagram labeled “Under a line-symmetry constraint,” the *left* and *right* panels represent nucleic acids with odd and even numbers of base pairs, respectively. In both cases, base pairs labeled with the same letter were edited using the same increment or decrement.

To avoid a combinatorial explosion of parameter patterns, two measures were introduced. First, line symmetry was imposed, reflecting the regularity observed in nucleic acid duplex structures. Second, a two-step workflow was implemented: (1) each parameter was modified individually to create models and perform a first round of MR, and patterns yielding favorable results were selected; (2) these selected patterns were then combined across Tilt, Roll, and Twist to create new models for a second round of MR.

All steps of our new method, as detailed in the Materials and Methods section and Figure 4B, were automated using shell scripts and integrated into a single software package named 4MRNA. 4MRNA is an abbreviation for “Massive Multi-type Model Molecular Replacement for Nucleic Acids,” with “4M” representing the four consecutive words beginning with “M,” “R” representing “Replacement,” and “NA” representing “Nucleic Acids.”

After a simple installation procedure, users can execute 4MRNA from the command line. The workflow is initiated by entering the command “4MRNA” in the directory containing the reflection file in MTZ format. Users are then prompted to select the nucleic acid type from A-form DNA, B-form DNA, or A-form RNA and to enter the sequence. Once these steps are completed, parameter adjustment, model generation, and subsequent MR are performed automatically within a single package. In short, users can run 4MRNA easily by preparing only the reflection file and basic nucleic acid information. Furthermore, to allow greater flexibility, the customize mode is also provided. In this version, a few shell scripts are provided separately rather than as a single package, but each script can be edited by the user. This is designed with advanced uses in mind, including the generation of more diverse models and the incorporation of models prepared by other methods for MR. Detailed usage instructions are provided in the Supplemental Methods.

The program has been publicly released to ensure accessibility and usability (https://github.com/S-Ando-Biophysics/4MRNA).

### Test cases

The validity of 4MRNA was examined using four test cases of nucleic acid duplexes: a standard duplex, a duplex containing a bulge-loop, duplexes containing internal-loops, and a nucleic acid containing hairpin-loops (Fig. 5). These four types collectively cover the principal classes of duplex structures and are described in detail in the following sections. In all cases, MR by 4MRNA was successfully performed, and the results are summarized in Supplemental Table S2. The number of created models and the computation time required for MR in each test case are provided in Supplemental Table S3.

**FIGURE 5.**
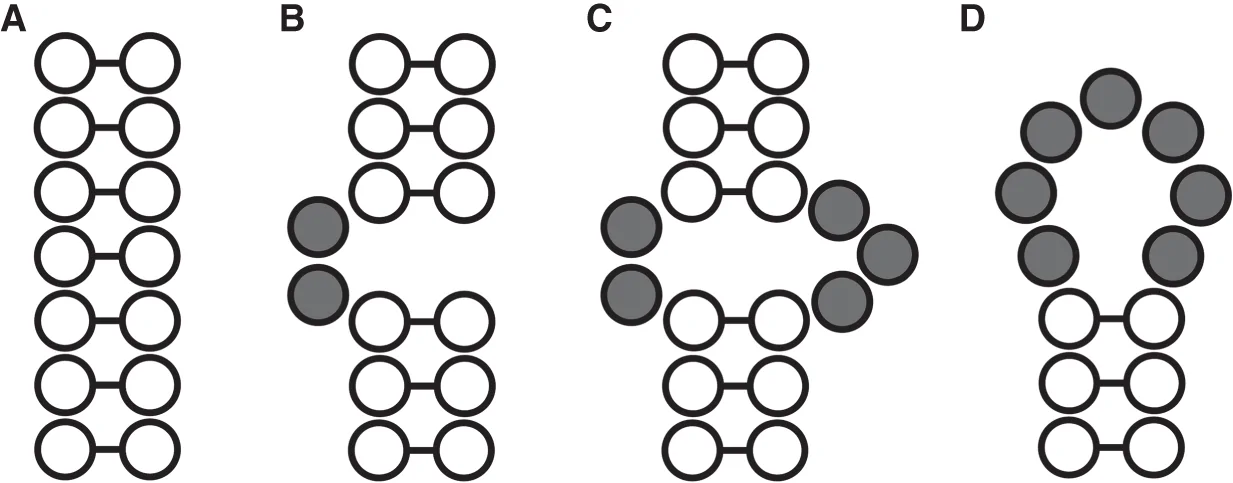
The type of duplex. (*A*) Standard duplex: A continuous helical region formed by canonical Watson–Crick A-T(U) and G-C base pairs between two complementary strands. (*B*) Bulge loop: One strand contains one or more nucleotides that are not base-paired due to the absence of complementary bases on the opposing strand. These unpaired bases typically adopt either a bulge-out conformation, protruding into the solvent, or a bulge-in conformation, stacking between adjacent base pairs. (*C*) Internal loop: Both strands contain one or more nucleotides that do not participate in canonical base-pairing, causing a local deviation from the regular helical geometry. These opposing unpaired regions may form noncanonical base pairs or remain unpaired. (*D*) Hairpin loop: A single strand folds back on itself to form a stem–loop structure. The stem is formed by intra-strand canonical base pairs, while the loop comprises nucleotides that may be unpaired, form noncanonical base pairs, or both.

### Test case 1: standard duplex

For the validation of a standard duplex, PDB ID 257D (Mooers et al. 1995) was used.

The MR was successful, with the best statistical values reaching LLG = 223 and TFZ = 11.8. Figure 6A shows the superimposition of the diverse models created under 4MRNA, whereas Figure 6B depicts the model yielding the best statistical values superimposed with the correct structure deposited in the PDB. The root-mean-square deviation (RMSD) for all atoms was 0.658 Å, indicating an excellent structural match. The parameter-change pattern that produced this optimal model is illustrated in Figure 6C.

**FIGURE 6.**
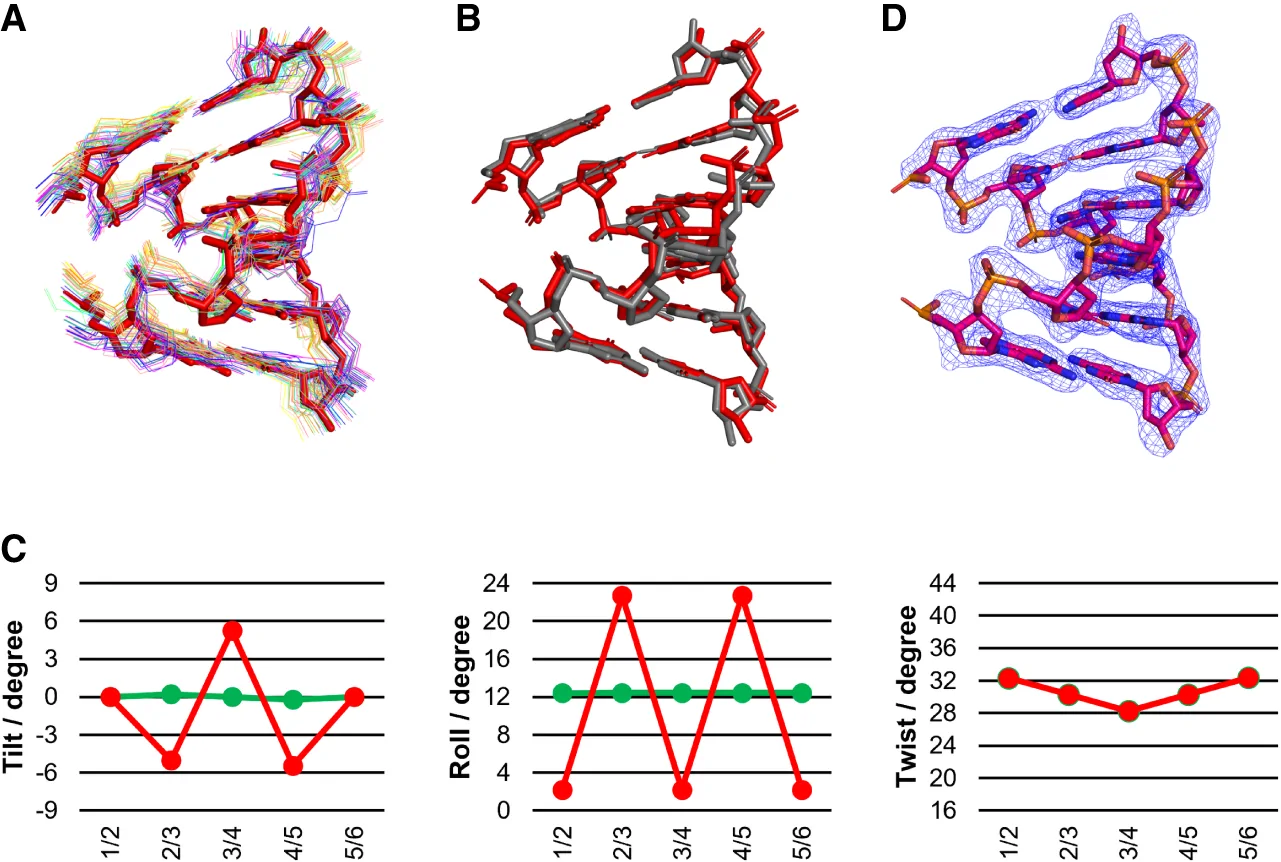
(*A*) A superimposition diagram of the diverse models created in the second round of 4MRNA. The best model is shown in red with stick representation, while the others are displayed as lines. (*B*) A superimposition diagram of the best model created under 4MRNA (red) and the correct structure registered in the PDB (gray) for the test case of a standard duplex. The RMSD for all atoms was 0.658 Å. (*C*) The parameter modifications of the model yielded the best results of MR (red). The horizontal axis represents the base-pair steps (in the format of upper base pair/lower base pair), while the vertical axis represents the parameter values. The green line represents the parameters of the ideal model generated using the program *fiber* in *3DNA*. (*D*) The electron density map (2mFo-DFc map) of 257D (blue) and the best model (after refining once; red). The 2mFo-DFc electron density map is contoured at a 1.0σ level.

Furthermore, Figure 6D displays the electron density map (2mFo-DFc map) of 257D together with the model after the first round of refinement. The refinement resulted in *R*-factor = 21.9% and *R*-free = 28.9%, suggesting that the structural analysis could be finalized with only a few additional steps, such as the placement of water molecules around the nucleic acid molecule.

In the original structure determination of 257D, the phase problem was solved by MR using an ideal A-form duplex as a search model. Therefore, for comparison, MR was also performed using an ideal A-form duplex model generated with the software *Coot* (Emsley et al. 2010). This approach also yielded a correct MR solution, with LLG = 146 and TFZ = 8.3, and provided an interpretable electron density map (Supplemental Fig. S7). Thus, although this conventional approach was sufficient to solve this standard duplex, the 4MRNA gave higher MR statistics, indicating that 4MRNA provided a better search model in this case.

### Test case 2: duplex containing a bulge loop

The PDB ID 1T0E (Shandrick et al. 2004) was used for the test case of a duplex containing a bulge loop. When loop regions are present, the structure is partitioned into multiple stem regions flanking the loop, and models are created for each stem separately. In this example, the sequence was partitioned into an 8-mer stem (long stem) and a 7-mer stem (short stem) (Fig. 7A).

**FIGURE 7.**
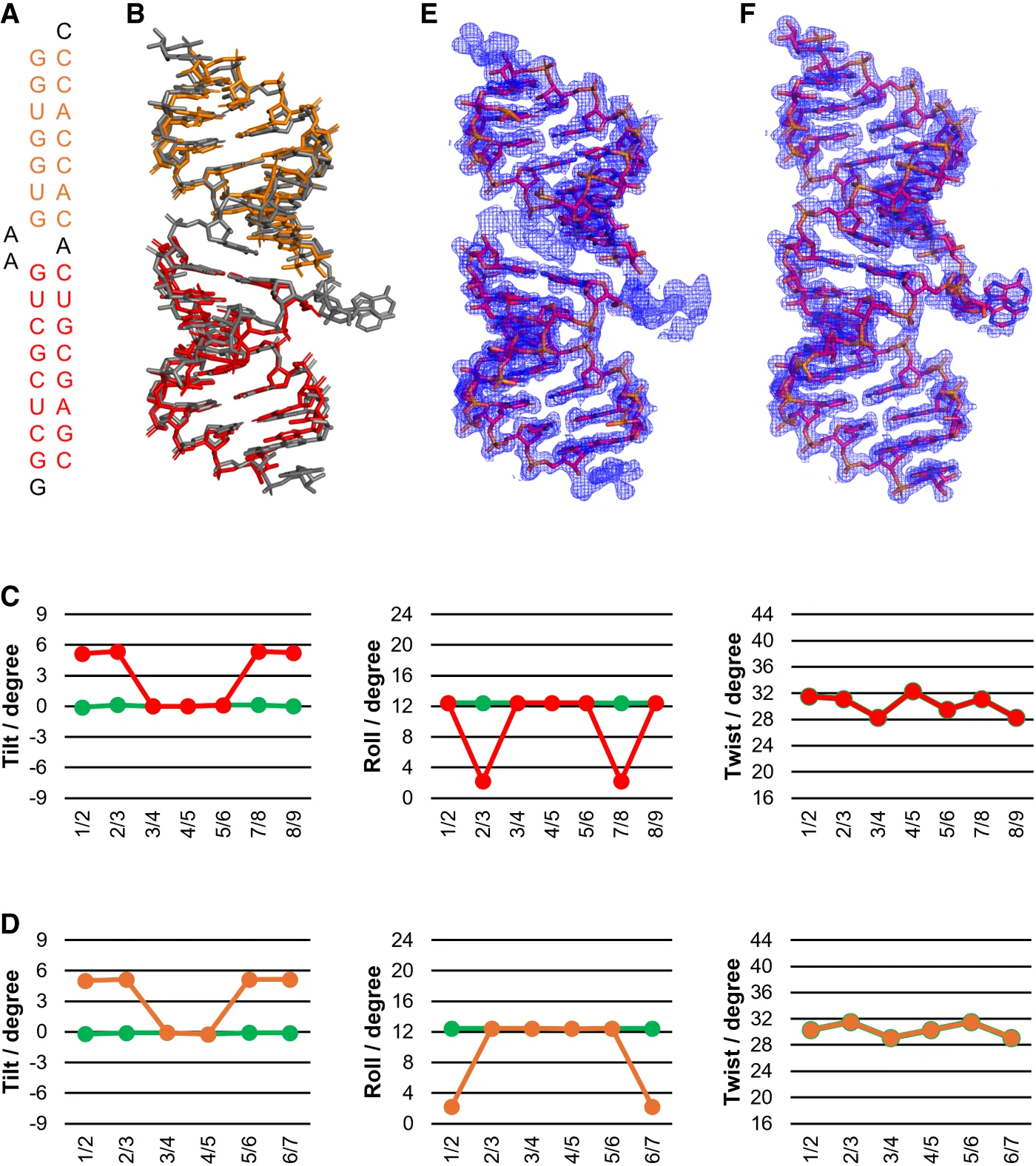
(*A*) The sequence of 1T0E. The sequence is designed to have one loop region, and the models of one long stem (red) and one short stem (orange) were created. (*B*) A superimposition diagram of the best created models and the correct structure registered in the PDB (gray) for the test case of a bulge loop. The long stem model is red, and the RMSD was 0.814 Å. The short stem model is orange, and the RMSD was 0.776 Å. (*C*) The patterns of parameter changes used to create the best long stem model (red). The horizontal axis represents the base-pair steps (in the format of upper base pair/lower base pair), while the vertical axis represents the parameter values. (*D*) The patterns of parameter changes used to create the best short stem model (orange). The horizontal axis represents the base-pair steps (in the format of upper base pair/lower base pair), while the vertical axis represents the parameter values. The green lines in *C* and *D* represent the parameters of the ideal model generated using the program *fiber* in *3DNA*. (*E*, *F*) The electron density map (2mFo-DFc map) of 1T0E is depicted in blue, while the best model is depicted in red. For (*E*), the model after one round of refinement is shown, whereas for (*F*), the model after placing nucleotides into the loop regions is shown. The 2mFo-DFc electron density maps are contoured at a 1.0σ level.

The MR was successful, with the best statistical values reaching LLG = 343 and TFZ = 15.3. In Figure 7B, the created models yielding the best statistical values are superimposed with the correct structure deposited in the PDB. The RMSDs for long and short stem regions were 0.814 and 0.776 Å, respectively, indicating an excellent structural match. The parameter patterns corresponding to the best models are shown in Figure 7C and D.

Moreover, the electron density map (2mFo-DFc map) of 1T0E and the model after the first round of refinement are shown in Figure 7E. This refinement resulted in *R*-factor = 36.0% and *R*-free = 38.3%. The bulge loop region was easily positioned manually, resulting in reduced *R* values (*R*-factor = 27.4% and *R*-free = 29.4%; Fig. 7F).

1T0E was also originally determined by MR using an ideal A-form duplex as a search model. To compare 4MRNA with this conventional approach, an ideal A-form duplex model was generated with the software *Coot* and used for MR. This approach yielded an MR solution with LLG = 118 and TFZ = 6.0. Although the resulting electron density map was interpretable, several regions did not fit the model appropriately, and the entire model was oriented in the opposite direction, requiring additional cycles of refinement and model correction (Supplemental Fig. S8). Thus, as also observed for 257D, this comparison demonstrates that 4MRNA can create search models closer to the correct structure. Furthermore, this comparison demonstrates that 4MRNA makes the subsequent refinement process more straightforward than with the conventional approach, because the models obtained with 4MRNA fitted the stem regions well from the initial MR solution.

### Test case 3: duplexes containing internal loops

To validate the case of nucleic acids containing internal loops, two structures having the same sequence but adopting different conformations were examined: PDB ID 3TD0 and PDB ID 3TD1 (Kondo 2012). In this case, the sequence was partitioned into one 8-mer stem (long stem) and two 5-mer stems (short stems) (Fig. 8A). The MR was successfully performed for both 3TD0 and 3TD1 using 4MRNA, despite their structural differences.

**FIGURE 8.**
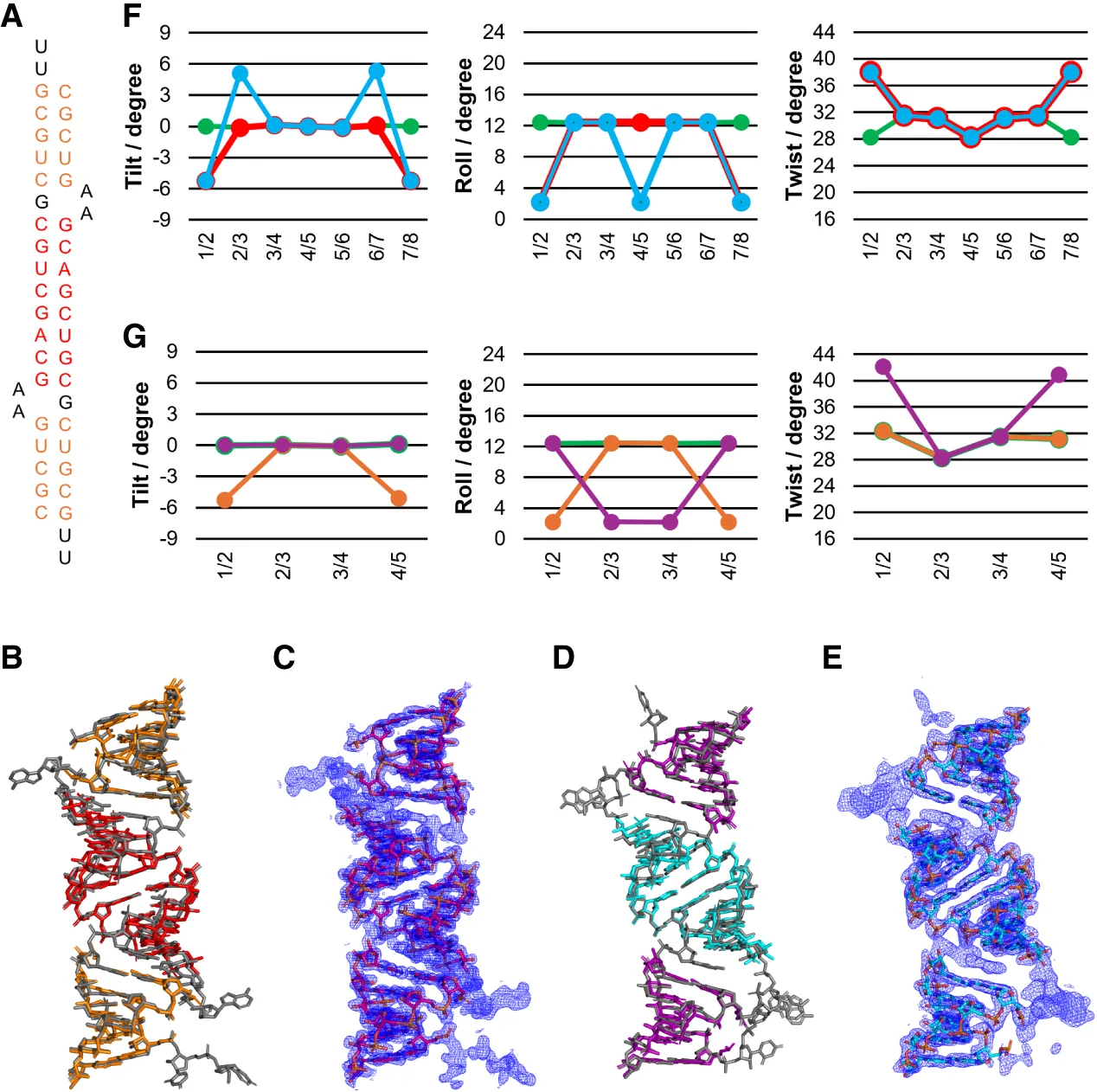
(*A*) The sequence of 3TD0 and 3TD1. These sequences are designed to have two loop regions, and the models of one long stem (red) and two short stems (orange) were created. (*B*) A superimposition diagram of the best models (red, orange) and the correct structure (gray) of 3TD0. The long stem model is red, and the RMSD was 0.789 Å. The short stem models are orange, and the RMSDs were 0.917 and 0.444 Å. (*C*) The electron density map (2mFo-DFc map) of 3TD0 (blue) and the best model (after refining once; red). The 2mFo-DFc electron density map is contoured at a 1.0σ level. (*D*) A superimposition diagram of the best models (cyan, purple) and the correct structure (gray) of 3TD1. The long stem model is cyan, and the RMSD was 0.534 Å. The short stem models are purple, and the RMSDs were 0.687 and 0.750 Å. (*E*) The electron density map (2mFo-DFc map) of 3TD1 (blue) and the best model (after refining once; cyan). The 2mFo-DFc electron density map is contoured at a 1.0σ level. (*F*) The patterns of parameter changes used to create the best long stem model for 3TD0 (red) and 3TD1 (cyan). The horizontal axis represents the base-pair steps (in the format of upper base pair/lower base pair), while the vertical axis represents the parameter values. (*G*) The patterns of parameter changes used to create the best short stem model for 3TD0 (orange) and 3TD1 (purple). The horizontal axis represents the base-pair steps (in the format of upper base pair/lower base pair), while the vertical axis represents the parameter values. The green lines in *F* and *G* represent the parameters of the ideal model generated using the program *fiber* in *3DNA*.

For 3TD0, the best statistics of MR were LLG = 390 and TFZ = 13.1. The best models, when superimposed on the correct structure, showed RMSDs of 0.789 Å for the long stem and 0.917 and 0.444 Å for the short stems (Fig. 8B). The first round of refinement resulted in *R*-factor = 42.3%, and *R*-free = 44.6% (Fig. 8C). For 3TD1, the best statistics of MR were LLG = 514 and TFZ = 16.4. The RMSDs were 0.534 Å for the long stem region and 0.687 and 0.750 Å for the short stem regions, respectively (Fig. 8D). After the first round of refinement, the values were *R*-factor = 37.2% and *R*-free = 43.1% (Fig. 8E).

Although the *R* values remain slightly high at that stage, the electron density map indicated that the loop regions can be easily determined by manual positioning as shown in Supplemental Figure S9, which reduced the *R* values. For 3TD0, the *R*-factor got 33.3% and the *R*-free got 37.7%. For 3TD1, the *R*-factor got 29.6% and the *R*-free got 33.9%. After arranging the loop, the structural analysis could be finalized with only a few additional steps, such as the placement of water molecules and ligands around the nucleic acid molecule (Supplemental Fig. S10).

Moreover, Figures 8F and G show an overlay of the parameter patterns corresponding to the best models for 3TD0 and 3TD1, revealing that the two structures exhibit distinct parameter patterns. In other words, the two different conformations could be distinguished by distinct parameter patterns, and successful MR was achieved accordingly. These results confirm that the parameter modulation in 4MRNA is valid and demonstrate the effectiveness of the strategy, whereby structural diversity among created models increases the probability of approximating the correct structure and thereby improves the success rate of MR.

3TD1 was originally solved by MR using PDB ID 1MWL (Vicens and Westhof 2003) as a search model, whereas 3TD0 was originally determined by MAD phasing. For 3TD0, MR was not successful when 1MWL, 3TD1, or combinations of ideal A-form duplex models were used as search models (Supplemental Fig. S11). Thus, 3TD0 represents a case in which conventional MR is difficult, even when related structures are available. When a sufficiently similar known structure is available, as in the case of 3TD1, conventional MR is undoubtedly effective. However, the 3TD0 case illustrates that such a suitable search model is not always readily available, particularly when nucleic acids with the same sequences adopt different conformations. The successful MR solution obtained for 3TD0 using 4MRNA therefore demonstrates the value of our new method as a broadly applicable strategy for nucleic acid structure determination. It also suggests that, when experimental phasing is difficult or impractical, 4MRNA can facilitate structure determination by MR if native diffraction data are available.

### Test case 4: more complex structure including hairpin loops (tRNA)

As an example of a nucleic acid duplex containing hairpin loops, transfer RNA (tRNA) with PDB ID 2TRA (Westhof et al. 1988) was employed. Generally, tRNA has four stems, the acceptor arm, the D arm, the anticodon arm, and the T arm, but the acceptor arm and the T arm can be regarded as a single continuous stem (Supplemental Fig. S12). Accordingly, models of three duplexes were created: the duplex consisting of the stems of the acceptor arm and the T arm (hereafter referred to as the AT duplex), the duplex of the D-arm stem (the D duplex), and the duplex of the anticodon arm (the A duplex). These duplex models were then combined and subjected to MR. However, in determining the A duplex, 4MRNA was customized to expand the parameter increments and decrements to −3σ, −2σ, −σ, ±0, +σ, +2σ, +3σ, because the default parameter range from −2σ to +2σ did not yield a satisfactory MR solution for the A duplex.

The model yielding the best statistical results (LLG = 269 and TFZ = 11.3), its parameters, and the electron density map (2mFo-DFc map) of 2TRA are shown in Figure 9. The RMSDs for the AT duplex, the D duplex, and the A duplex were 0.962, 0.626, and 1.012 Å, respectively (Fig. 9A). Figure 9B shows the electron density map (2mFo-DFc map) of 2TRA and the model after the first round of refinement. After this refinement, the values were *R*-factor = 35.7% and *R*-free = 40.9%. As in other cases, the loop regions could be easily positioned manually, and the *R* values were reduced (*R*-factor = 14.9% and *R*-free = 19.7%) (Supplemental Fig. S13).

**FIGURE 9.**
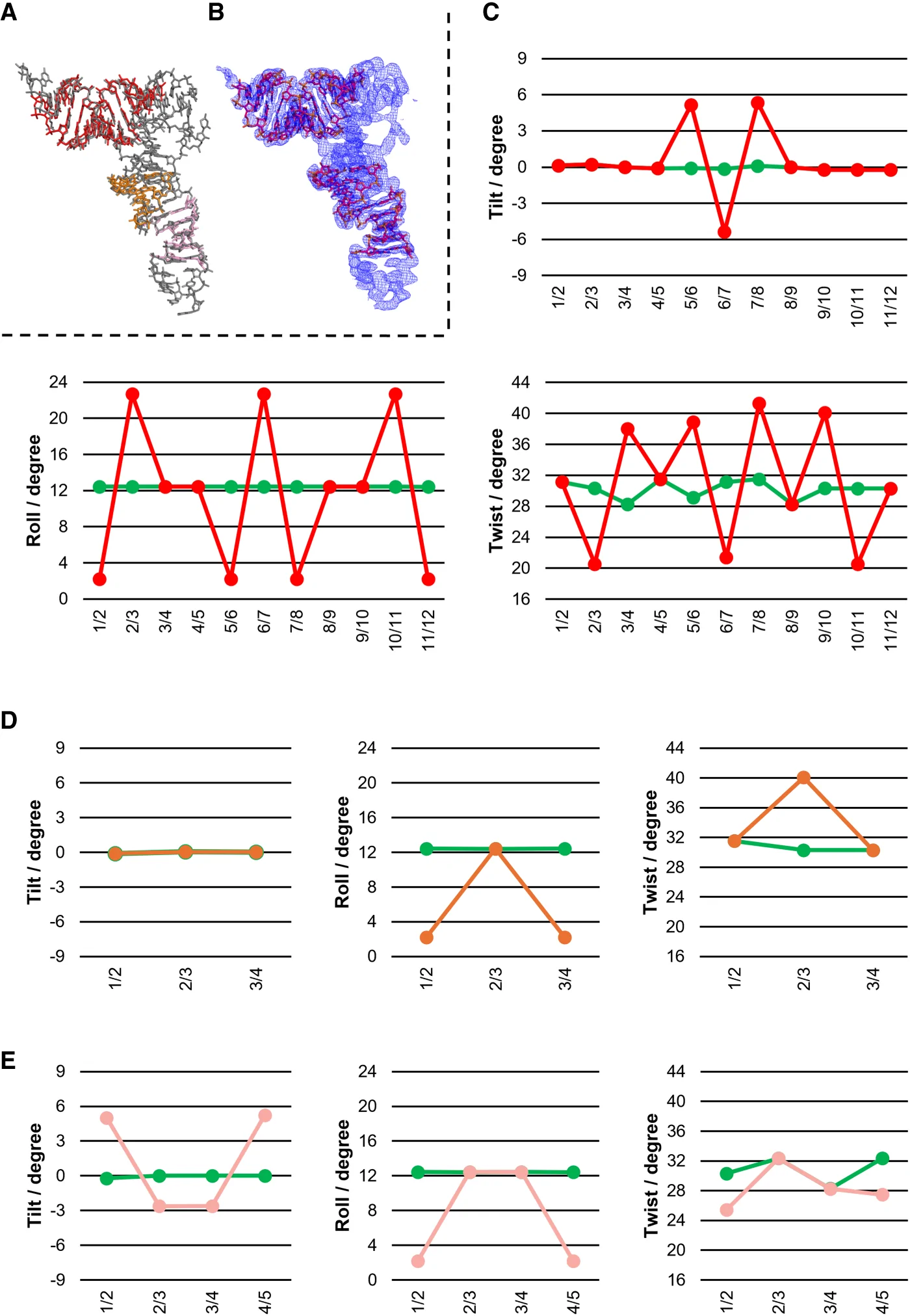
(*A*) The best created model (red, orange, pink) superimposed on the correct structure registered in the PDB (gray). The AT duplex model is red, and the RMSD was 0.962 Å. The D duplex model is orange, and the RMSD was 0.626 Å. The A duplex model is pink, and the RMSD was 1.012 Å. (*B*) The electron density map (2mFo-DFc map) of 2TRA (blue) and the best model (after refining once; red). The 2mFo-DFc electron density map is contoured at a 1.0σ level. (*C*) The patterns of parameter changes used to create the best AT duplex model (red). The horizontal axis represents the base-pair steps (in the format of upper base pair/lower base pair), while the vertical axis represents the parameter values. (*D*) The patterns of parameter changes used to create the best D duplex model (orange). The horizontal axis represents the base-pair steps (in the format of upper base pair/lower base pair), while the vertical axis represents the parameter values. (*E*) The patterns of parameter changes used to create the best A duplex model (pink). The horizontal axis represents the base-pair steps (in the format of upper base pair/lower base pair), while the vertical axis represents the parameter values. The green lines represent the parameters of the ideal model generated using the program *fiber* in *3DNA*.

The original structure determination of 2TRA was performed using multiple isomorphous replacement (MIR), which is an experimental phasing method based on heavy atom derivatives, because MR had not yet developed at that time. If MR is attempted using conventional approaches, other known tRNA structures would first be tested as a search model. When PDB ID 6TNA (Sussman et al. 1978) was used as a search model representing other tRNA structures, no satisfactory MR solution was obtained, with LLG = 15 and TFZ = 4.5, and the resulting model showed poor agreement with the electron density map (Supplemental Fig. S14A). Therefore, a second strategy was next tested, in which the tRNA molecule was divided into several fragments, corresponding models were prepared for each fragment, and these models were used as separate search models. This approach was carried out in two ways, one using only stem models and the other using models that included the hairpin loop regions (Supplemental Fig. S14B). In the first way, three stem models corresponding to the AT duplex, D duplex, and A duplex were generated as ideal A-form duplexes using the software *Coot*. In the second way, the same ideal A-form duplex models were used for the AT duplex and D duplex, whereas the model of the entire anticodon arm was used instead of the A duplex model. The anticodon arm model was prepared by extracting the anticodon loop region from 6TNA and extending the two strands with an ideal A-form duplex generated using the software *Coot*. In both ways, the MR statistics (LLG and TFZ) were low, and interpretable electron density maps were not obtained (Supplemental Fig. S14C,D).

Taken together, this test case demonstrates that 4MRNA can enable successful MR even for targets that are difficult to solve using conventional approaches.

### New structure determined by 4MRNA

To further demonstrate the effectiveness and practicality of 4MRNA, structure determination of a previously unreported target was attempted. The target was a self-complementary DNA with the sequence 5′-GGGTGCTXCCC-3′ (X = 2-aminopurine), and the asymmetric unit was estimated to contain four molecules (eight strands). Since this sequence was entirely new, no suitable related search model was available. In addition, MR trials using a model generated by AlphaFold3 or an ideal A-form duplex model did not yield a satisfactory solution (Supplemental Fig. S15). Therefore, 4MRNA was applied to this target.

Structure determination was successfully achieved using 4MRNA. The model yielding the best statistical results yielded LLG = 409 and TFZ = 13.5, and the parameter patterns of the best model are shown in Figure 10A. After the first round of refinement, the *R*-factor and *R*-free were 39.6% and 44.4%, respectively, and the electron density map (2mFo-DFc map) is shown in Figure 10B. Although the *R* values after the first round of refinement were relatively high, the loop regions were clearly visible in the electron density map. After manual placement of these regions (Fig. 10C), the *R*-factor and *R*-free decreased to 24.9% and 30.3%, respectively. When the best MR solution was superimposed on the final refined structure (*R*-factor = 21.7% and *R*-free = 26.3%), the RMSD was 1.265 Å (Fig. 10D,E).

**FIGURE 10.**
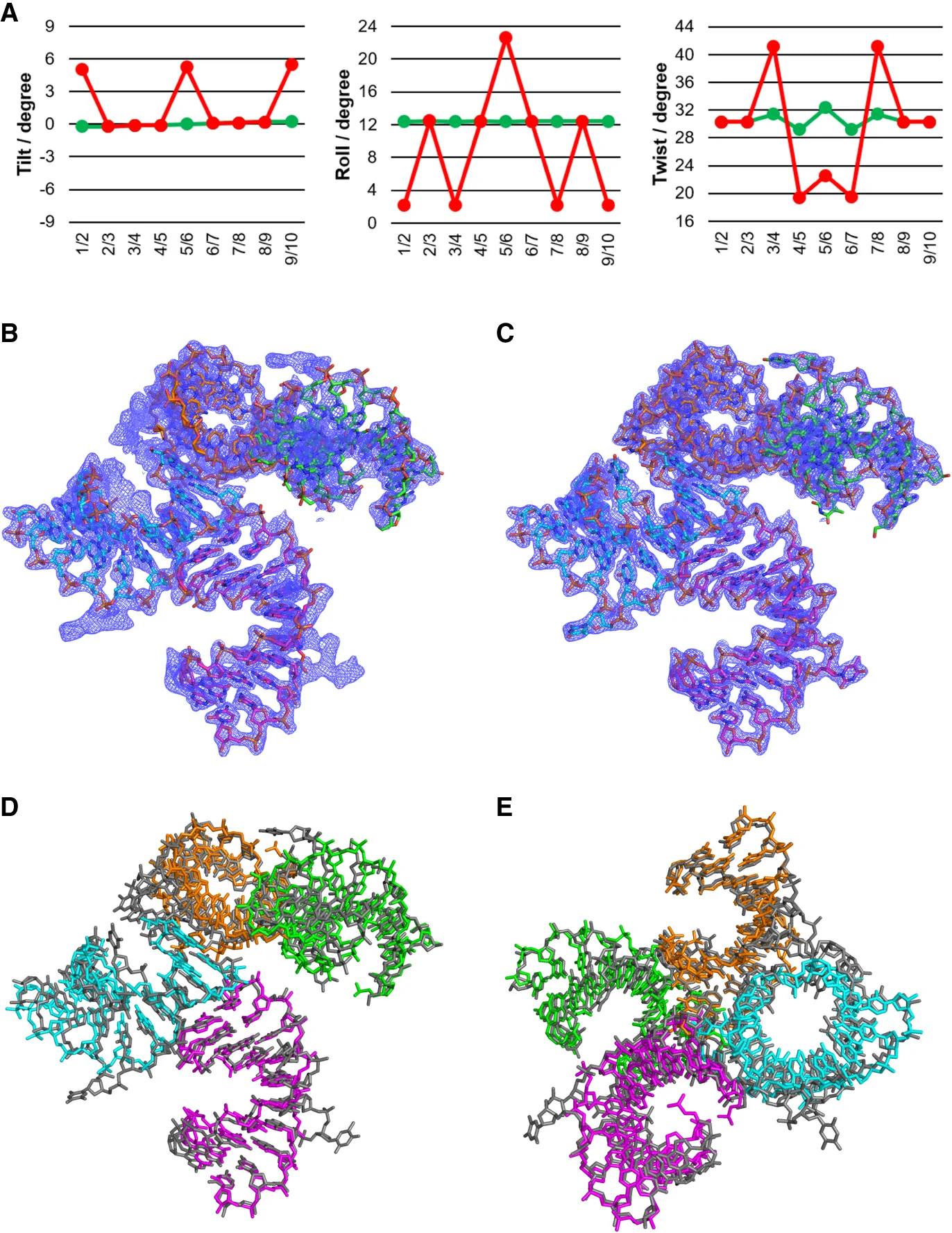
(*A*) The parameter modifications of the model yielded the best results of MR (red). The horizontal axis represents the base-pair steps (in the format of upper base pair/lower base pair), while the vertical axis represents the parameter values. The green line represents the parameters of the ideal model generated using the program *fiber* in *3DNA*. (*B*) The electron density map (2mFo-DFc map; blue) and the best models (after refining once). The 2mFo-DFc electron density map is contoured at a 1.0σ level. The best models are colored by molecules (magenta, cyan, green, and orange). (*C*) The structure and electron density map (2mFo-DFc map) after manual placement of nucleotides in the loop region. (*D*, *E*) Superimposition diagrams of the best MR model and the final refined structure viewed from two directions. The final refined structure is shown in gray, whereas the four molecules in the best MR result are shown in different colors. The overall RMSD was 1.265 Å.

The final coordinates were deposited in the PDB under the accession code 25LU. Detailed crystallographic statistics, including crystal data, data-collection statistics, and structure-refinement statistics, are summarized in Supplemental Table S4.

## DISCUSSION

This study represents the first detailed investigation into how helical parameters with large standard deviations affect structure determination by MR. It was found that varying Buckle, Propeller, and Opening had little effect on MR results, whereas adjustments to Tilt, Roll, and Twist markedly enhanced MR performance. This difference suggests that the success of MR is influenced primarily by global structural information, such as base-pair stacking (captured by inter-base-pair parameters), rather than by local deformations within individual base pairs (captured by intra-base-pair parameters).

Indeed, by adjusting the values of Tilt, Roll, and Twist in various patterns, models that closely matched the correct structures could be created in advance, as demonstrated in Figures 6B, 7B, 8B,D, and 9A, thereby enabling successful MR. If more detailed investigations are required, particularly when default increments or decrements (−2σ, ±0, +2σ) do not yield successful phase determination, 4MRNA can be customized, as exemplified by test case 4, to expand the parameter variation range (−3σ, −2σ, −σ, ±0, +σ, +2σ, +3σ) and thereby produce additional models that improve the likelihood of success.

The effectiveness of 4MRNA was validated not only for simple duplexes but also for more complex structures containing loops and even for tRNA which represents a highly intricate nucleic acid fold. For highly complex nucleic acid structures such as tRNA, it is effective to first identify stem regions using secondary-structure prediction tools (Sato et al. 2021). Based on this information, the structure can be treated under the assumption that multiple stem segments are connected to form one continuous stem, as demonstrated in this study.

Furthermore, in the test case of duplexes containing two internal loops, MR was successfully achieved for both structures using distinct parameter patterns. The second molecule (3TD1) is known to represent a distorted conformation induced by binding of an aminoglycoside to the first molecule (3TD0) (Kondo 2012). These results therefore provide compelling evidence that different conformational states can be resolved by MR using appropriately diversified parameter patterns, strongly supporting the validity of the parameter-modulation strategy introduced here.

Notably, the test cases examined in this study highlight the broad applicability of 4MRNA. For example, PDB ID 1T0E, which contains a bulge loop, has provided key structural insights into fluorescence labeling of RNA (Shandrick et al. 2004), while PDB IDs 3TD0 and 3TD1 are directly linked to structure-based drug design (SBDD) of nucleic acid targeting therapeutics (Kondo 2012). Thus, the ability of 4MRNA to handle such diverse structures demonstrates not only its utility as a tool for fundamental structural studies but also its potential impact on applied research.

The source code for this approach has been made publicly available on GitHub (https://github.com/S-Ando-Biophysics/4MRNA). The version implemented as a single command allows the entire 4MRNA pipeline to be run from start to finish, lowering the barrier for users without extensive coding expertise and thereby promoting broad adoption of the method. In parallel, a customizable mode is provided as a more advanced option, capable of addressing highly complex cases such as test case 4.

In conclusion, the novel strategy proposed in this study has proven highly effective. Given that it has successfully enabled the determination of new structures that could not be solved by conventional approaches, 4MRNA holds strong potential to become an indispensable tool for nucleic acid crystallography. 4MRNA is expected to open new avenues for applications in structure-based drug design and nanotechnology based on nucleic acid structures by accelerating fundamental research on nucleic acid structures.

## MATERIALS AND METHODS

### Statistical analysis of helical parameters

The analysis was performed using the program *Analysis* in *Web 3DNA* (Li et al. 2019). The analysis targeted all nucleic acid crystal structures in which two strands form an A-form or B-form double helix in the asymmetric unit, deposited in the PDB as of May 2024, totaling 371 A-form duplexes and 463 B-form duplexes. In previous studies, the data set was restricted to structures with a resolution of 2.0 Å or better (Olson et al. 2001). However, since it is common to perform structure determination by MR even for diffraction data at lower resolutions, no such restriction was imposed in this analysis. In addition, the number of entries in the PDB has increased since that study. Therefore, it was possible to analyze a larger data set than those used in previous studies.

Outliers were removed by the Smirnov–Grubbs test (two tailed) at a significance level of 0.05, with the rejection criteria determined using an R script (Supplemental Script S1). This test iteratively selects the value farthest from the mean and excludes it as an outlier if it satisfies the criterion described in Equation (1), repeating the procedure until no further outliers remain. The right-hand side of Equation (1) was calculated using the R script, where *n* represents the sample size and *t* denotes the critical value of the *t*-distribution with *n*−2 degrees of freedom.

(1) $$\frac{\left|\mathrm{value}-\mathrm{mean}\right|}{\mathrm{standard}\;\mathrm{deviation}}>\frac{(n-1)t}{\sqrt{n(n-2)+nt^{2}}}$$

### Narrowing down key helical parameters through further analysis of the effect on molecular replacement

The impact of six parameters (Buckle, Propeller, Opening, Tilt, Roll, and Twist) on MR was examined. For this validation, a 6-mer DNA structure with PDB ID 1IH1 (Vargason et al. 2001) was used.

Using the program *Fiber* in *Web 3DNA*, the ideal duplex model was generated from the input sequence. The structure was then analyzed with the program *Analysis* in *Web 3DNA*, and the resulting parameter values were exported as text files. The text files were modified to vary the parameters of each base pair at one of three levels (−3σ, ±0, +3σ). For intra-base-pair parameters, each of the 6 bp was independently adjusted at three levels, resulting in 729 (=3^6^) models for each parameter. For inter-base-pair parameters, each of the 5 bp steps was independently adjusted at three levels, resulting in 243 (=3^5^) models for each parameter.

The modified files were uploaded to the program *Rebuild* in *Web 3DNA* to generate the corresponding structural models. MR was performed using *Phaser* (McCoy et al. 2007) for each model. The Python script used for automated model generation is provided in Supplemental Script S2, and the shell script used for MR is provided in Supplemental Script S3.

### Analysis of the regularity of key helical parameters

The values of Tilt, Roll, and Twist were calculated using the program *Analysis* in *Web 3DNA*. The analysis was performed on all nucleic acid duplex crystal structures, including 371 A-form duplexes and 463 B-form duplexes. For each duplex, the parameter values were plotted as graphs, where the horizontal axis represents the stack number and the vertical axis indicates the parameter values. In each plot, the data points were connected by lines to visualize the parameter regularity across the duplex.

### Development of a new approach

In 4MRNA, the three helical parameters identified as most influential for MR, Tilt, Roll, and Twist, were systematically varied. Each parameter was adjusted using three increments or decrements (−2σ, ±0, +2σ), a choice motivated by the statistical principle known as the 68-95-99.7 rule. According to this rule, assuming a normal distribution, 68.27% of the data falls within ±1σ of the mean, 95.45% within ±2σ, and 99.73% within ±3σ. In other words, most of the structural diversity can be explained by variations in parameters within the range of ±2σ from the mean, even in cases of significant structural distortions.

However, for a duplex containing *n* base pairs, each of the *n*−1 base-pair steps can be adjusted at three levels for a given inter-base-pair parameter, resulting in 3^*n*−1^ possible combinations. When three inter-base-pair parameters (Tilt, Roll, and Twist) are varied simultaneously, the total number of combinations becomes (3^*n*−1^)^3^ = 27^*n*−1^, which rapidly becomes prohibitively large even for high-performance computing. To make the workflow practical, the entire process of model generation followed by MR was designed as a two-stage workflow. The first stage was a screening process in which the three key parameters, Tilt, Roll, and Twist, were individually varied to generate models, each of which was subjected to MR. The second stage was a brute-force combination process in which the parameter patterns that yielded favorable MR results in the first stage were combined across the three parameters to create new models, which were again subjected to MR.

An outline of the model-building process in the first round is as follows. To further reduce the number of models, a line-symmetry constraint was imposed. The molecule was divided in half at its center, and base pairs equidistant from the center were required to share identical patterns of increase or decrease. Under this constraint, if *n* is odd, there are $3^{\frac{n-1}{2}}$ possible variations for each parameter, and if *n* is even, there are $3^{\frac{n}{2}}$ possible variations for each parameter. This relationship can be expressed more generally as $3^{\left\lfloor \frac{n}{2}\right\rfloor }$ using the floor function. Since there are three parameters, the total number of models in the first round becomes $3\times \;3^{\left\lfloor \frac{n}{2}\right\rfloor }=3^{\lfloor \frac{n}{2}\rfloor +1}$.

1. The program *fiber* in *3DNA* (Lu and Olson 2008) was used to input the sequence of a target nucleic acid and generate an ideal duplex structure based on average helical parameter values or values closely approximating the average.
2. The ideal model was analyzed using the programs *find_pair* and *analyze* in *3DNA* to extract precise helical parameter values for each base pair. These data were saved as a .par file for further modifications.
3. Parameters for each base pair were systematically adjusted in three increments or decrements (−2σ, ±0 [no change], and +2σ) with a line-symmetry constraint to create .par files with many structural variations.
4. These edited .par files were input into the program *rebuild* in *3DNA* to generate a diverse set of models corresponding to each parameter pattern. In addition, the backbone geometry was optimized using *phenix.geometry_minimization* from the *Phenix* suite as in the program *Rebuild* in *Web 3DNA* (Liebschner et al. 2019; Li et al. 2019).

These steps were automated using shell scripts. The resulting models were then subjected to MR with the program *Phaser*. Since MR had to be iterated many times, its execution was also automated using shell scripts.

The model construction process in the second round was as follows.

1. For each parameter, up to seven patterns were selected from the first round based on favorable MR statistics. The selection criteria were the top two ranked by LLG, the top two ranked by TFZ, and the top three ranked by the product of LLG and TFZ, with overlaps reducing the total to a minimum of three. These criteria were empirically established during the development of the new approach through iterative validation on representative test cases.
2. The selected patterns were simultaneously combined across the three parameters, and corresponding parameter .par files were created.
3. These edited .par files were input into the program *rebuild* in *3DNA* to generate models corresponding to each parameter pattern. The backbone geometry was optimized using *phenix.geometry_minimization* from the *Phenix* suite.

This procedure yielded between 27 (=3 × 3 × 3) and 343 (=7 × 7 × 7) models. The entire process was automated using shell scripts for model construction and for repeated MR calculations, in the same manner as in the first round. The MR results obtained in this step were regarded as the final outcomes of the new strategy.

The detailed workflow described above is also summarized in Figure 4B.

### Validation of effectiveness

To verify the effectiveness of the new strategy, phase determination was performed for four types of known structures and one unreported structure.

For each test case of known structures, the nucleotide sequence was obtained from the PDB and the corresponding structure factor file was obtained from the PDB-REDO (Joosten et al. 2014). Based on the sequence information, models were created using 4MRNA, and MR was performed with the acquired structure factor file for each model. The effectiveness of the strategy was then evaluated by comparing the best MR solutions with the reference structures deposited in the PDB. The PDB IDs are as follows: the standard double helix is 257D, the duplex containing a bulge loop is 1T0E, the duplexes containing internal loops are 3TD0 and 3TD1, and tRNA is 2TRA.

When 4MRNA was applied to test cases containing loop regions, the structure was partitioned into multiple stem regions flanking the loop, and models were created separately for each stem. The structural determination by MR was then carried out in a stepwise manner as follows. For instance, if the structure can be divided into two stems, A and B, multiple diverse models of stem A, created by adjustment of the parameters in 4MRNA, were used together with the averaged model of stem B in iterative MR calculations to determine the structure of stem A. Subsequently, the determined structure of stem A was combined with multiple diverse models of stem B, and MR was repeated. As a result, the structures of both stems A and B were determined. In this procedure, the averaged model corresponds to the ideal model generated with the program *fiber* at the beginning of the 4MRNA workflow.

For the tRNA case, which represents a duplex with hairpin loops, the customizable mode of 4MRNA was employed. The procedure was as follows: (1) MR was performed using multiple AT duplex models created by 4MRNA in combination with the ideal D duplex model to determine the AT duplex structure; (2) MR was then conducted using the determined AT duplex model together with multiple D duplex models created by 4MRNA to determine the D duplex structure; and (3) MR was finally performed using multiple A duplex models created by 4MRNA in combination with the determined models of the AT duplex and D duplex to determine the A duplex structure. In addition, during the determination of the A duplex, the default parameter ranges from −2σ to +2σ was first tested. However, this range did not yield a satisfactory MR solution. Therefore, the parameter increments and decrements were expanded to −3σ, −2σ, −σ, ±0, +σ, +2σ, and +3σ in order to cover a wider parameter range and create more diverse models. Since the default range was set to ±2σ based on the 68-95-99.7 rule, expansion to ±3σ was adopted as a natural next step to increase model diversity.

For the unreported structure, a self-complementary DNA with the sequence 5′-GGGTGCTXCCC-3′ (X = 2-aminopurine) was employed. Based on the Matthews coefficient (Matthews 1968) calculated from the unit cell parameters and space group obtained after data processing with the software *XDS* (Kabsch 2010), the asymmetric unit of the crystal was estimated to contain four molecules. Since the sequence was entirely novel, no suitable search models for MR were available among previously reported structures in the PDB. A model generated by AlphaFold3 was also tested for MR, but no solution was obtained. In addition, an ideal A-form duplex model generated with the software *Coot* gave LLG = 233 and TFZ = 8.7. However, only three molecules were placed although four molecules were expected in the asymmetric unit, and the models showed poor agreement with the electron density map (Supplemental Fig. S15). Therefore, 4MRNA was applied for structure determination.

After obtaining the initial phases by MR, the first round of refinement was performed using *phenix.refine* from the *Phenix* suite (Afonine et al. 2012; Liebschner et al. 2019), in order to verify whether the structure had been correctly determined and to assess whether further refinement steps could be pursued. For structures containing loops, further refinement was undertaken by manually placing bases into the loop regions. For this modeling, the structure was superimposed with the electron density map using the software *Coot*. Additional refinement cycles were subsequently performed to evaluate whether the *R* values decreased.

## SUPPLEMENTAL MATERIAL

Supplemental material is available for this article.

## Figures and Tables

**Shin Ando:**
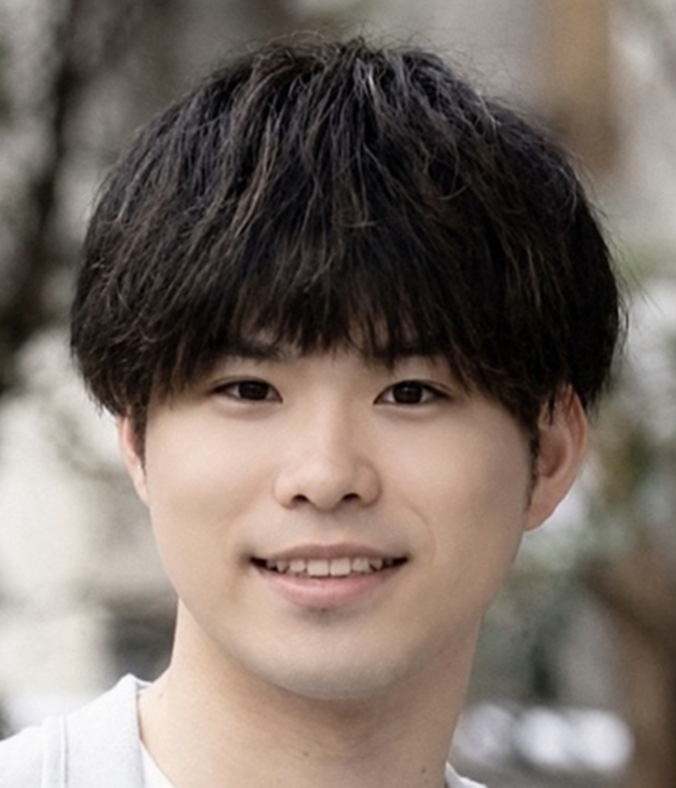


## MEET THE FIRST AUTHOR

***Meet the First Author(s)* is an editorial feature within *RNA*, in which the first author(s) of research-based papers in each issue have the opportunity to introduce themselves and their work to readers of *RNA* and the RNA research community. Shin Ando is the first author of this paper, “4MRNA: a new approach for nucleic acid molecular replacement using models with diverse parameter patterns.” Shin is a PhD student in the Biophysics Laboratory led by Professor Jiro Kondo at Sophia University, Japan. His research focuses on elucidating nucleic acid structures at the atomic and molecular levels using biophysical approaches such as X-ray crystallography, with the goal of applying structural insights to the design of new nucleic acid therapeutics.**

**What are the major results described in your paper and how do they impact this branch of the field?**

We developed 4MRNA, a new method for improving molecular replacement in nucleic acid crystallography. We identified key structural parameters and their regularities that strongly influence the results of molecular replacement, and used this knowledge to propose a strategy for creating appropriate model sets. We also implemented software that fully automates both model generation and molecular replacement calculations. 4MRNA has the potential to become an essential tool in nucleic acid X-ray crystallography and will help to greatly accelerate structural studies of nucleic acids.

**What led you to study RNA or this aspect of RNA science?**

I first became interested in nucleic acid structures when I was in high school, and joined my current laboratory as an undergraduate student to study the crystal structures of a wide variety of nucleic acids. During this work, I sometimes encountered cases in which X-ray diffraction data had been collected, but phase determination remained difficult. These experiences made me realize the need for strategies designed specifically for nucleic acids, because many conventional methods were developed primarily for proteins.

**What are some of the landmark moments that provoked your interest in science or your development as a scientist?**

When I was 5 years old, my mother was diagnosed with a rare disease for which no effective treatment was available. This experience led me to decide that I wanted to become a scientist who could develop new technologies that contribute to treatment through entirely new approaches. By reading scientific books and attending seminars, I became interested in structure-based drug design and nucleic acid therapeutics during my first year of high school. Since then, I have wanted to pioneer a research field that combines these two areas. I therefore sought an environment where this would be possible and joined Professor Kondo's laboratory, one of the few laboratories in the world pursuing this kind of research.

**What are your subsequent near- or long-term career plans?**

My current research, including the PhD work that I recently started, mainly focuses on fundamental studies involving the structural analysis of a wide variety of nucleic acids. In the near term, I would like to continue solving nucleic acid structures and deepen our understanding of their structural principles. At the same time, I hope to apply the knowledge gained from these fundamental studies to more applied research. In particular, I aim to pursue the ambitious goal of designing nucleic acid therapeutics based on structural information. To achieve this long-term goal, I would like to pursue an academic career as a researcher.
